# Editorial: New trends and approaches in perioperative pharmacotherapy, volume III

**DOI:** 10.3389/fphar.2024.1453007

**Published:** 2024-07-19

**Authors:** S. Soghomonyan, S. D. Bergese, N. Stoicea

**Affiliations:** ^1^ Department of Anesthesiology, The Ohio State University Wexner Medical Center, Columbus, OH, United States; ^2^ Department of Anesthesiology, Stony Brook University Renaissance School of Medicine, Stony Brook, NY, United States; ^3^ Solid Biosciences Inc., Charlestown, MA, United States

**Keywords:** perioperative care, pharmacotherapy, anesthesia, surgical complications, postoperative recovery

The perioperative period increases the existing stress for patients and healthcare providers and raises many problems for physicians preparing the patient for surgery. Preoperative patient stress may arise from a fear of the risks associated with surgery and anesthesia, an unfamiliar medical environment, cost of therapy, pain, and even death (Ji et al., 2022).

Responsibilities of the clinician include helping the patient to overcome the perioperative stress and anxiety, optimizing the drug therapy to keep the comorbid conditions under control, and taking all the necessary measures to decrease the risks of surgery. One of the important determinants of success remains the effective perioperative pain control based on opioid and non-opioid therapy, maintenance therapy, regional anesthesia, and enhanced recovery measures developed for specific surgical interventions (Hill and Lefkowits, 2021).

Nowadays, physicians encounter more patients with complex comorbidities requiring preparation in a short time to safely undergo surgery. Until recently, many of those cases would be considered inoperable requiring prolonged treatment prior to surgery. The safe and effective management of the perioperative period for such patients requires continuous update in medical knowledge, familiarity with recent advances in pharmacotherapy, and drug delivery methods. The perioperative clinician must consult various sources, from expert consensus statements to randomized studies, to make informed recommendations for preoperative medication management (Sahai et al., 2022).

Advances in surgery mandate corresponding adaptation of the perioperative treatment strategies to help the patients tolerate the procedure avoiding unnecessary risks and discomfort.

Our previously completed and published Research Topic and it's update on perioperative pharmacotherapy proved to be well accepted with over 95,000 views and downloads, and multiple citations. It became obvious that a new update is necessary to present the recent developments and trends in the field of perioperative pharmacotherapy.

With that in mind, our editorial team readily accepted an invitation from the journal to prepare a new update of the Research Topic. With a meticulous selection process, ten manuscripts were included in the Research Topic covering various important aspects of perioperative care.

Wondesen Tsige et al. studied the appropriateness of stress ulcer prophylaxis (SUP) strategies in university hospital settings in Ethiopia. The authors found out inappropriate use of SUP in many surgical patients and concluded that this may be an important area for multidisciplinary research to develop standardized protocols for effective SUP.

Shelton et al. present two interesting cases and discuss the use of buprenorphine micro-dosing in surgical patients with opioid use disorder. The method suggested by the authors helps to avoid the risk of precipitated withdrawal and at the same time effectively manage the surgical pain in this complex patient group.

Echeverria-Villalobos et al. present an interesting review discussing the interconnection between the activation of glial and immune cells leading to creation of a neuroinflammatory state, which, in turn, promotes development of acute and chronic pain.

Mazzotta et al. present a mini review discussing the mechanisms of development, prophylaxis and treatment of the postoperative sore throat (POST). Being one of the most common post-anesthesia adverse events, POST is not well studied, and new approaches and treatment options are necessary for its effective management. The authors suggest a new intubation technique with rotation of the endotracheal tube prior to removal of the stylet, which may decrease the impact on the tracheal wall and decrease the incidence of POST.

Elias et al. discuss the role of dopamine receptor antagonist antiemetics, particularly Amisulpride, in management of postoperative nausea and vomiting (PONV). Given the widespread use of 5-HT3 antagonists for PONV prophylaxis and hypothesized side effects of the older dopamine receptor blockers, Amisulpride is suggested as an effective rescue antiemetic to treat persistent PONV.

Xu et al. present a meta-analysis of preclinical studies which studied the role of vagus nerve stimulation (VNS) in attenuating myocardial ischemia-reperfusion injury–a cascade of events undermining the protective benefits of revascularization, contributing to ventricular dysfunction, and increasing mortality. The authors conclude that VNS can effectively limit infarct expansion, ventricular remodeling, cardiac dysfunction, and improve the left ventricular ejection fraction.

Ginosyan et al. discuss the perioperative management strategies of patients with antiphospholipid and catastrophic antiphospholipid syndromes undergoing urgent neurosurgical procedures. The authors discuss the use of plasmapheresis, blood component and clotting factor transfusions in preparation of patients to surgery. They also discuss the potential risk of clotting complications in this extremely challenging group of patients.

Soghomonyan et al. present an interesting patient case of progressive portal, mesenteric, and splenic venous thrombosis. The authors highlight the necessity of timely establishment of diagnosis based on imaging studies and initiation of multi-component therapy with anticoagulants, anti-inflammatory drugs, and antibiotics. Such approach helps to stop thrombus progression, prevent irreversible intestinal ischemia, and allows for re-canalization of the occluded veins.

Liu et al. present the results of their clinical and *in vivo* experiments to find an association between the increased plasma renin concentration and activity caused by direct vasodilators, which promote progression of the abdominal aortic aneurysm. The authors present their interesting data indicating that directly acting antihypertensive drugs hydralazine and minoxidil increase the circulating renin concentration and activity, increase the aortic wall degeneration, and promote progression of abdominal aortic aneurysm.

Wang et al. conducted a network meta-analysis and reviewed the short-term antithrombotic strategies after left atrial appendage occlusion (LAAO). They compared dual antiplatelet therapy (DAPT), direct oral anticoagulants (DOACs), and vitamin K antagonist therapy (VKA) in patients who had experienced LAAO. Their study results indicate that there is no significant difference between DAPT, DOACs, and VKA in terms of stroke, device-related thrombosis (DRT), and major bleeding events after LAAO. DAPT was ranked the worst among all antithrombotic strategies due to the higher risk of stroke, DRT, and major bleeding events, while VKAs were ranked the preferred antithrombotic strategy. However, DOACs, according to authors, are worthy of consideration due to their advantage of convenience.

We believe the manuscripts included in the Research Topic will be interesting to the practitioners involved in perioperative patient care and will help them optimize their therapeutic plans suitable for their patient needs.
